# Rotor Termination Is Critically Dependent on Kinetic Properties of *I*_Kur_ Inhibitors in an *In Silico* Model of Chronic Atrial Fibrillation

**DOI:** 10.1371/journal.pone.0083179

**Published:** 2013-12-20

**Authors:** Eberhard P. Scholz, Paola Carrillo-Bustamante, Fathima Fischer, Mathias Wilhelms, Edgar Zitron, Olaf Dössel, Hugo A. Katus, Gunnar Seemann

**Affiliations:** 1 Department of Internal Medicine III, University Hospital Heidelberg, Heidelberg, Germany; 2 Institute of Biomedical Engineering, Karlsruhe Institute of Technology (KIT), Karlsruhe, Germany; 3 Theoretical Biology and Bioinformatics, Utrecht University, Utrecht, The Netherlands; 4 German Centre for Cardiovascular Research (DZHK) partner site Heidelberg/Mannheim, Heidelberg, Germany; Gent University, Belgium

## Abstract

Inhibition of the atrial ultra-rapid delayed rectifier potassium current (*I*_Kur_) represents a promising therapeutic strategy in the therapy of atrial fibrillation. However, experimental and clinical data on the antiarrhythmic efficacy remain controversial. We tested the hypothesis that antiarrhythmic effects of *I*_Kur_ inhibitors are dependent on kinetic properties of channel blockade. A mathematical description of *I*_Kur_ blockade was introduced into Courtemanche-Ramirez-Nattel models of normal and remodeled atrial electrophysiology. Effects of five model compounds with different kinetic properties were analyzed. Although a reduction of dominant frequencies could be observed in two dimensional tissue simulations for all compounds, a reduction of spiral wave activity could be only be detected in two cases. We found that an increase of the percent area of refractory tissue due to a prolongation of the wavelength seems to be particularly important. By automatic tracking of spiral tip movement we find that increased refractoriness resulted in rotor extinction caused by an increased spiral-tip meandering. We show that antiarrhythmic effects of *I*_Kur_ inhibitors are dependent on kinetic properties of blockade. We find that an increase of the percent area of refractory tissue is the underlying mechanism for an increased spiral-tip meandering, resulting in the extinction of re-entrant circuits.

## Introduction

Atrial fibrillation (AF) is the most common sustained arrhythmia in the elderly and is associated with serious health consequences. In search for novel therapeutic strategies, much effort has been made to identify pharmacological targets for an atrial specific anti-arrhythmic drug therapy [1], [2]. Therapeutic strategies that either restore the conduction velocity (CV) or increase action potential duration (APD) are thought to be effective in terminating fibrillatory activity and maintaining sinus rhythm [3]. Because the ultra-rapidly activating delayed rectifier current (*I*_kur_) is virtually absent in human ventricles but highly expressed in the atria, it has been proposed to be a promising target for atrial-selective antiarrhythmic therapy [4]. Indeed, several studies show that pharmacological *I*_Kur_ inhibition affects atrial action potential (AP) configuration and prolongs APD in trabeculae from AF atria [5], [6]. Therefore, much effort has been devoted to identifying selective pharmacological inhibitors of human *I*_Kur_ current.

Although selective *I*_Kur_ inhibition theoretically represents an effective mechanism in AF therapy, clinical data are somewhat conflicting. Olson and co-workers reported a loss-of-function mutation within the Kv1.5 potassium channel, the molecular basis of human *I*_Kur_, as the cause of a form of familial AF [7]. Furthermore, pharmacological inhibition of *I*_Kur_ has been associated with both anti- and pro-arrhythmic effects on human atrial cells [6], [8]–[13]. These obvious inconsistencies might be explained by individual kinetic properties of the different *I*_Kur_ inhibitors, including time- and voltage-dependence of block as well as simultaneous inhibitory effects on other cardiac ion channels [14].

Using a mathematical model of chronic atrial fibrillation (cAF) we first analyzed the influence of time- and voltage-dependence of *I*_Kur_ inhibition on basic electrophysiological tissue properties. A simplified two-dimensional *in silico* model of cAF was then applied to test the hypothesis that functional reentry and termination of AF is influenced by the kinetic properties of inhibitory compounds. Our results show that antiarrhythmic properties of *I*_Kur_ inhibitors are strongly dependent on the ability to prolong the wavelength thereby reducing the percent area of non-refractory space.

## Models

### Mathematical model description

The Courtemanche, Ramirez, Nattel (CRN) model [15] is a mathematical model of the AP based on ionic current data obtained from human atrial cells. It describes the electrical behavior of the cell with a set of nonlinear-coupled ordinary differential equations that reconstruct ion concentrations, ionic currents, bindings to intracellular structures, and the transmembrane voltage.

The occurrence and perpetuation of AF can be favored by changes in the electrical, structural and contractile function of the atria, a process termed “atrial remodeling”. Electrical remodeling is commonly promoted by AF itself and is thought to be due to the high rate of atrial activation [16]. Alterations caused by electrical remodeling mainly relate to altered ion current densities and can be integrated into the CRN model by adapting the maximal conductance of the affected channels to fit the experimentally observed changes [17]. In short, the implemented changes are an increase of *I*_K1_ by 110%, and a decrease of *I*_Ca,L_ and *I*_to_ by each 65%. Additionally, the tissue conductivity was reduced by 30% in order to reflect gap junction remodeling.

### Implementation of time and voltage dependence of *I*_Kur_ blockade

The formulation of *I*_Kur_ described by the CRN model is given by
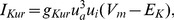
(1)where g_Kur_ is the maximum conductance, u_a_ is the activation gating variable, u_i_ is the inactivation gating variable, V_m_ is the transmembrane voltage, and E_K_ is the Nernst voltage. To simulate the human atrial AP with voltage- and time-dependent block of *I*_Kur_, the equation was modified according to Tsujimae et al. [14] by multiplying a factor that describes the non-blocked fraction of *I*_Kur_:
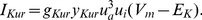
(2)The non-blocked fraction y_Kur_ was calculated with the following differential equation
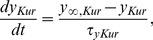
(3)where y_∞,Kur_ is the steady state value of y_Kur_ and τ_yKur_ is the time constant. y_∞,Kur_ describes the voltage dependence of the blockade and is expressed by a Boltzmann function with a half-maximum block voltage of −40 mV and a slope of 5 mV
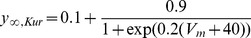
(4)By this way, a form of blockade was described where *I*_Kur_ is not inhibited at hyperpolarized voltages whereas at large depolarized voltages 90% of the channels can be blocked. The time dependence of the *I*_Kur_ blockade is described by
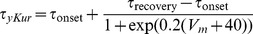
(5)where τ_onset_ is the time constant for the activation of channel inhibition and τ_recovery_ the time constant for recovery from inhibition. Since different agents seemed to block the channel preferentially in the open state, τ_onset_ describes the time constant at large depolarized voltages. In contrast, channels recover from inhibition after repolarization. Therefore, τ_recovery_ describes the time constant at large hyperpolarized voltages. A variation of both parameters results in a different time-dependence of the blockade.

### Tissue simulations

In order to simulate excitation propagation, the monodomain model was used. It was implemented in the simulation framework acCELLErate [18] in which the Rush–Larsen scheme for gating variables and a forward Euler scheme for the remaining ODEs was used. To discretize the spatial equations, the finite difference method was applied with a time increment of 10 µs and a space step of 0.1 mm. For the one-dimensional investigations, e.g. effective refractory period (ERP) and CV restitution, we used a tissue strand of 20 mm×0.1 mm×0.1 mm. For the analysis of the CV of the waveback, a tissue strand of 60 mm×0.1 mm×0.1 mm was used. The two-dimensional tissue patch to calculate fibrillatory waves was composed of 100 mm×100 mm×0.1 mm. We adapted the isotropic conductivity for the tissue simulations to obtain a CV of ≈750 mm/s at a basic cycle length (BCL) of 1 s in the normal case.

APD, ERP, CV and WL restitution were calculated as described in [19]. To initiate fibrillatory waves in the 2D tissue patch, a specific stimulation protocol was applied. First, the left side of the patch was stimulated for 4 beats with a BCL of 0.3 s to obtain a plane wave. Subsequently, a cross-field stimulus in the lower half of the patch was applied during a time where half of the patch remained still in the refractory period of the last plane wave activation. This created a new excitation front into the left half of the patch but not into the right one. This broken excitation front quickly developed into a spiral wave. To split the spiral wave in many rotors, several impulses were set at different places each in the front of the excitation. With this stimulation protocol 5 stable rotors could be initiated in the case of cAF. The trajectories of the spiral cores were tracked with an algorithm presented in [19].

### Percent area of refractory tissue

The percentage of refractory tissue was determined by retrieving the value of the h-variable of the sodium current:
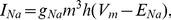
(6)of each cell. Depending on the state of the inactivation gate, the h-value varies between values of 0 and 1. For our analysis, cells were defined as refractory when the h-value was below a cutoff of 0.5. This threshold has shown a good correlation with excitability in human atrial tissue (unpublished data).

### Frequency analysis

The extracellular electrical potential Φ_e_(P,t) at a position P in an infinite homogeneous space can be approximated by:
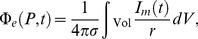
(7)where *I*_m_ is the electrical source current and r the distance from the position P to the current source. This approximation assumes that the electrical conductivity of both the extracellular space, and the medium external to the tissue equals to σ.

The pseudo-ECG was calculated as the difference between the signals at positions P1 and P2, both situated in the center of the 2D patch 10 mm apart from each other and with a distance of 5 mm from the tissue,
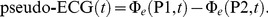
(8)By calculating the power spectral density of the pseudo-ECG using the Fast Fourier Transform, the dominant frequency (DF) can be determined as the peak in the signal.

## Results

### Electrophysiological characteristics of atrial tissue under AF-induced remodeling

**Figure 1:**
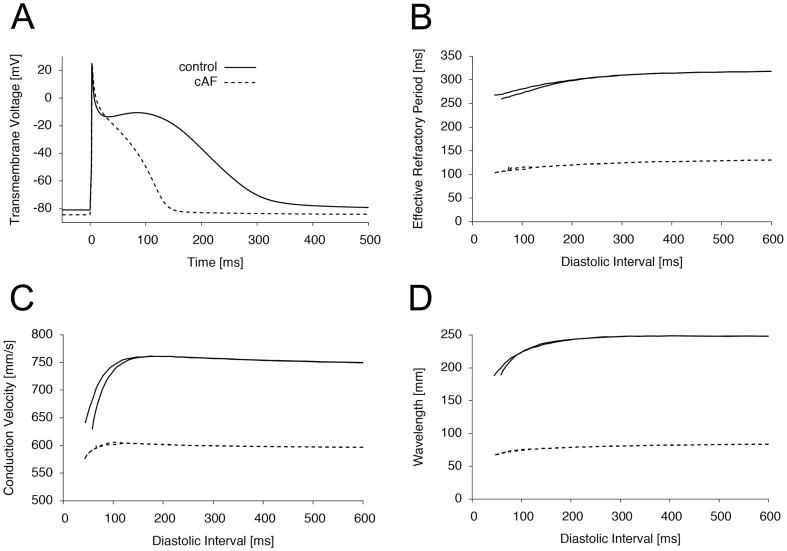
Electrophysiological properties of cAF tissue. (A) Simulated AP recordings of isolated cells from normal (solid line) and remodeled atrial tissue (dashed line). (B) Restitution of ERP under physiological conditions (solid lines) and cAF (dashed lines). (C, D) CV and WL restitution for physiological conditions (solid lines) and cAF (dashed lines).

The aim of this study was to dissect antiarrhythmic mechanisms of different kinetic properties of *I*_Kur_ inhibition on functional reentry and AF termination using an *in silico* model of atrial tissue. To create a model of chronic AF, we first integrated the electrophysiological remodeling processes into the cellular model [17]. Compared to the physiological situation, cAF cells showed distinct electrophysiological properties, similar to those observed in human experimental data [20]. In line with previous data [14], we show that the AP shape is severely altered in cAF cells, exhibiting a triangulation and profound shortening. For example, the APD at 30, 70 and 90% of repolarization (APD_30_, APD_70_ and APD_90_) were reduced from 140 ms, 230 ms, and 300 ms in normal atrial cells to 40 ms, 100 ms, and 129 ms in cAF cells, respectively (Figure 1A). To further assess the frequency dependence (restitution) of the ERP, CV and wavelength (WL), we extended our analysis to one-dimensional tissue strips at increasing pacing rates. In line with the results obtained in the single cell simulations the ERP exhibited a severe reduction in cAF atrium (Figure 1B). At a diastolic interval of 500 ms, the ERP was reduced from 320 ms to 137 ms. In agreement with human data, electrophysiological remodeling also resulted in a reduction of the CV [21]. In our simulations, the CV was reduced from 761 mm/s to 598 mm/s at a diastolic interval of 500 ms (Figure 1C). Since the WL is the product of ERP and CV, the effects of atrial remodeling on the WL were even more pronounced. At the same diastolic interval, the WL was reduced from 243 mm to 82 mm in the cAF tissue, resulting in a higher susceptibility for the occurrence and maintenance of multiple spiral waves (Figure 1D).

### Pharmacological properties of *I*_Kur_ blockade

**Figure 2:**
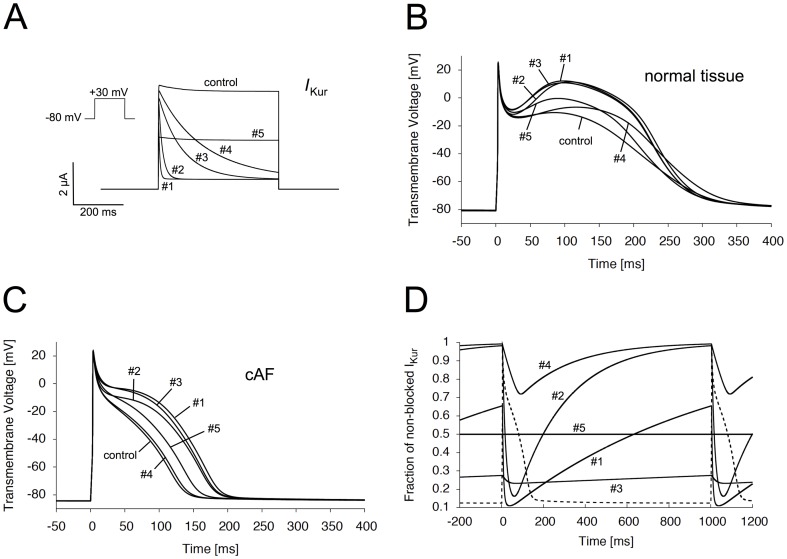
Pharmacological properties of simulated *I*_Kur_ inhibition. (A) Effect of inhibition on *I*_Kur_ elicited by a simulated voltage step (500 ms) to +30 mV (see inset). (B) Effect of the different inhibitory model compounds on AP trajectories in cells from normal atrial tissue. (C) Depending on the kinetic properties, *I*_Kur_ inhibition resulted in an elevation of the plateau potential (#1, #2, and #3) as well as AP prolongation (#1, #2, #3, and #5) in cells from cAF tissue. (D) Effects of onset and recovery time constants on the non-blocked fraction of *I*_Kur_ blockade established at a pacing rate of 1 Hz. The underlying AP derived from a cAF cell is depicted in a dashed line.

*In vivo* and *in silico* experiments have demonstrated the ability of *I*_Kur_ inhibitors to prolong APD in cAF cells [6]. However, the AP modification strongly depends on the pharmacological characteristics of channel blockade [14]. To analyze the association between the time- and voltage dependence of *I*_Kur_ block and its antiarrhythmic efficacy, we established a set of five different model compounds of *I*_Kur_ block (Table 1). Whereas compound #1 represents a fictitious *I*_Kur_ blocker with putative ideal antiarrhythmic properties on the basis of the publication by Tsujimae et al. [14], compound #2 and #3 relate to kinetics of diphenyl phosphine oxides (DPO), a recently described group of potent *I*_Kur_ inhibitors [14], [22]. Compound #4 was included to simulate slow onset and fast recovery kinetics, as an opposite situation of #1. Compound #5 represents a tonic blockade, mimicking a loss-of-function mutation. Figure 2A displays the pharmacological effects of the different model compounds on a simulated current trace elicited by a rectangular voltage step applied with a frequency of 1 Hz (see inset). When applied to normal atrial cells, *I*_Kur_ inhibition had marked effects on the AP trajectory, especially within the plateau phase (Figure 2B). However, none of the model compounds caused a relevant prolongation of the terminal phase of the atrial AP. These results are in line with experimental data showing that the effect of *I*_Kur_ inhibition on APD is strongly dependent on the degree of electrical remodeling [6]. As expected, atrial APD was prolonged for most of the model compounds when tested in cAF tissue (Figure 2C). Whereas compound #1, #2, and #3 resulted in a relevant prolongation of the terminal repolarization phase (APD_90_), compounds #1 and #3 additionally elevated the potential of the plateau phase (Table 2). This particular effect was reflected by a strong modification of APD_30_ by compound #1 and #3, resulting in a prolongation from 40 ms to 110 ms and 103 ms, respectively. These findings agree with previously published data on simulated *I*_Kur_ inhibition in remodeled atrial tissue [23]. Furthermore, as proposed by Tsujimae et al. [14], the overall AP prolongation showed a strong dependence on pharmacological properties, with the most pronounced prolongation resulting from compounds with slow time constants of recovery (Table 1). By analyzing time course of unblocked *I*_Kur_ during a simulated AP at a heart rate of 1 Hz, we found that compound #2 and #4 accomplished a complete dissociation from the channel during the diastolic interval, whereas compound #1 and #3 accumulated (Figure 2D). This effect might explain the profound effect of the slowly dissociating compounds on atrial AP in both normal, and cAF cells (Figure 2B, C).

**Table 1:** Kinetic properties of *I*_Kur_ inhibitors.

Compound	τ_onset_ (ms)	τ_recovery_ (ms)	Properties
#1	5	1000	fast onset, slow recovery
#2	16	238	fast onset, fast recovery
#3	100	16000	slow onset, slow recovery
#4	200	250	slow onset, fast recovery
#5	n/a	n/a	tonic block

**Table 2:** Differential effect of *I*_Kur_ inhibitors on action potential configuration in remodeled atrial tissue.

Compound	APD_30_ (ms)	APD_70_ (ms)	APD_90_ (ms)
Control	40	100	129
#1	110	156	182
#2	91	146	174
#3	103	150	176
#4	43	105	134
#5	66	122	150

### Electrophysiological properties of remodeled tissue under pharmacological inhibition

**Figure 3:**
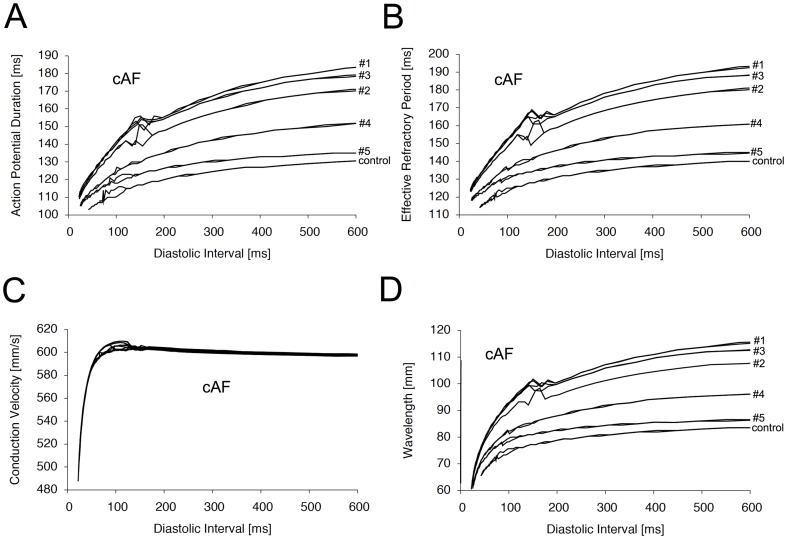
Electrophysiological properties of remodeled atrial tissue under *I*_Kur_ inhibition. Restitution curves of the APD (A), ERP (B), CV (C), and WL (D) under different types of *I*_Kur_ inhibition. Whereas APD, ERP, and WL restitution exhibits a strong dependence on kinetic properties of *I*_Kur_ inhibitors, the CV is not affected.

**Figure 4:**
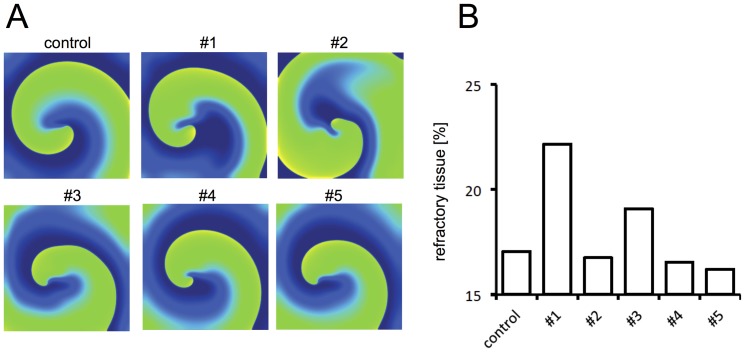
Percent area of refractory tissue. (A) Spiral wave activity of a single rotor under control cAF conditions and after inhibition with all five test compounds displayed at the end of the 5^th^ second. The transmembrane voltage is color-coded with blue representing −80 mV and yellow representing 0 mV. (B) Summarized data of the mean percentage of refractory tissue quantified over the last 4 seconds of inhibition (control: 17.2%, #1: 22.2%, #2: 16.8%, #3: 19.1%, #4: 16.6%, #5: 16.2%).

We next analyzed the pharmacological effects of the *I*_Kur_ blockade in dependence of the diastolic interval (restitution curve). With decreasing diastolic intervals, the APD exhibited a decrease for cAF control tissue, as well as all of the inhibitory model compounds (Figure 3A). Again, the most pronounced effect could be observed for compound #1 and #3 exhibiting slow unbinding characteristics. Below a specific diastolic interval, the APD of even and odd beats started alternating between two distinct values. This well-recognized phenomenon, termed electrical alternans, has been associated with the steepness of the restitution curve and might predispose to wavebreak [24], [25]. Indeed, in our experiments electrical alternans occurred earlier in cases of steep restitution curves (compounds #1, #2 and #3). However, wavebreak could not be observed in any of our 2D simulations (Fig. 4A). Analyzing the minimum diastolic interval, we find that compared to the control condition (42 ms) all test compounds resulted in a decrease of the minimum diastolic interval to values of 23 ms (#1), 24 ms (#2), 22 ms (#3), 26 ms (#4), and 25 ms (#5). These results suggest that the ERP is quite close to the APD. As expected from these results, the ERP restitution curve of the control condition and all test compounds strongly resembled the APD restitution (Fig. 3B). Next, effects of *I*_Kur_ inhibition on the restitution of the CV were analyzed. As expected from a pure inhibitor of repolarizing potassium current, no marked effect on the CV restitution could be observed (Figure 3C). Furthermore, using a long (6 cm) 1D tissue strip, effects of the different compounds on the waveback speed were analyzed. We find that the CV of the waveback did not differ from the CV of the wavefront (data not shown). The stability and occurrence of reentrant circuits was strongly dependent on the WL. Since the CV was identical for all model compounds in our simulations, the restitution curve of the WL strongly resembled the APD and ERP restitution, exhibiting the most pronounced WL prolongation for compound #1 and #3 (Fig. 3D).

### Tissue simulation and analysis of spiral waves

The electrophysiological effects of every *I*_Kur_ inhibitor were next analyzed in a simplified two-dimensional simulation of chronic remodeled atrium. We first analyzed whether the observed WL prolongation is reflected by an increase in the percent area of refractory tissue. For this purpose, a single rotor was induced using a simple cross-field stimulation protocol. Inhibition was started after 1 second and the rotor was followed over a time period of 5 seconds. Figure 4A displays the activation pattern at the end of the simulation for the control condition and all test compounds. The percent area of refractory space was determined by retrieving the value of the inactivation variable (h) of the sodium channel from all cells. Cells exhibiting a h-value greater or equal to 0.5 were defined as excitable. The mean percent area of refractory tissue during the last 4 seconds of inhibition is depicted in Fig. 4B. Under control conditions, the mean percent area of refractory space revealed 17.1% (Fig. 4B). Comparable values could be obtained for compound #2 (16.8%), #4 (16.6%), and #5 (16.2%) (Fig. 4B). Of note, the slightly lower values of these test compounds compared to the control conditions might be due to decreased spatial stability of the rotors. Interestingly, refractoriness was increased to 22.2 and 19.1% for compound #1 and #3, respectively. These values correspond well with the pronounced effect of these compounds on the wavelength (Fig. 3D).

**Figure 5:**
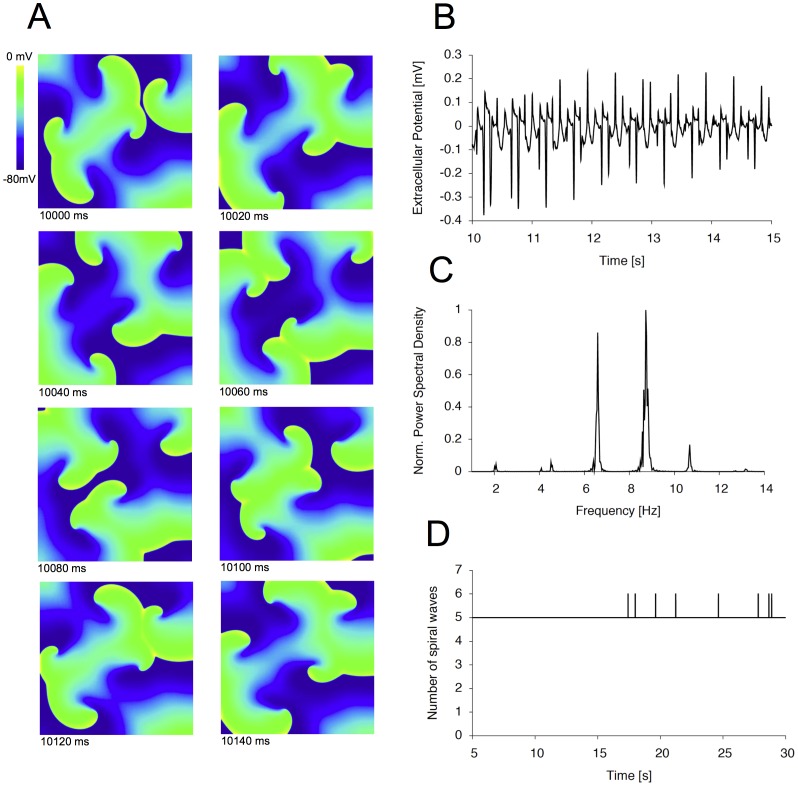
Automated analysis of spiral wave activity. (A) Sequence of images (in 20 ms steps) of spiral wave activity under control cAF conditions starting at the 10^th^ second. The transmembrane voltage is color-coded with blue representing −80 mV and yellow representing 0 mV. (B) Exemplary time interval (10^th^ to 15^th^ second) of the simulated unipolar pseudo-electrocardiogram (pseudo-ECG) recorded at the center of the atrial layer under control conditions. (C) Normalized power spectral density of atrial activation derived from the pseudo-ECG. (D) Number of rotors automatically detected by our analysis algorithm.

Next, effects of the compounds on multiple wavelets were studied. In our remodeled atrial model, a number of five rotors could be easily induced within an area of 100 cm^2^ using a cross-field stimulation protocol with additional stimuli under control conditions [17]. Of note, no stable rotor could be induced in our two-dimensional model of atrial tissue lacking electrophysiological remodeling (data not shown). This might be mainly attributed to the relation between the wavelength and the size of the 2D model and is in line with previous studies showing that stable rotors can only be induced in more realistic 3D models [26]–[28]. The electrical activity of the tissue sample was followed over a period of 30 seconds. Figure 5 depicts representative activation patterns under control conditions (cAF without compound). To further analyze the electrical activity we calculated a pseudo-ECG (Figure 5B). For this purpose, two virtual electrodes were placed 5 mm distant from the tissue, from which potential changes were measured. Using Fast Fourier Transformation, the dominant frequencies in the power spectral density could be obtained (Figure 5C). Under control conditions, a dominant frequency was detected at 6.59 and 8.73 Hz, corresponding to dominant frequencies obtained from ECGs of AF patients [29]. Next, we used an automated algorithm (see methods section) to automatically track the number of spiral waves. We found that under control conditions the initially established number of 5 rotors persisted over the complete time interval with only minor changes until the end of the simulation (Figure 5D).

### Rotor stability under pharmacological *I*_Kur_ inhibition

**Figure 6:**
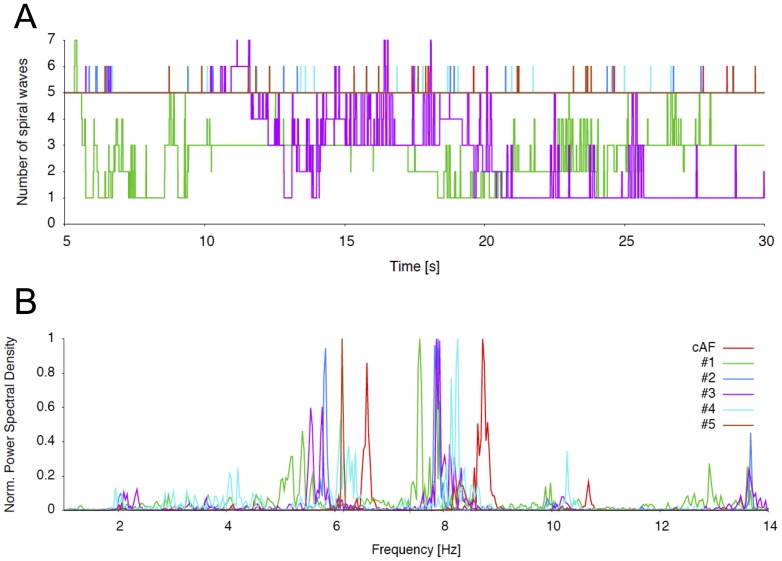
Rotor analysis under pharmacological *I*_Kur_ inhibition. (A) Number of spiral waves followed over a time period of 30 seconds. (B) Normalized power spectral density of electrical activity in remodeled tissue under control conditions and under *I*_Kur_ inhibition.

To analyze antiarrhythmic properties, rotors were induced in a tissue probe of cAF tissue as described above. As soon as five stable rotors had established (after 4 s), the inhibitory model compounds were added one at a time. The effects on the number of spiral waves were again followed over a time period of 30 seconds. Under control conditions, the number of 5 spiral waves persisted over the complete time period (Figure 6A). Similar results were obtained for compounds #2, #4 and #5 (Figure 6A). In contrast, the number of spiral waves was effectively reduced to 3 and 1 rotor after 30 seconds, when adding the inhibitory compounds #1 and #3, respectively (Figure 6A).

Wavelength prolongation has been shown to reduce rotor frequency by enlarging the reentrant circle [30]. In agreement with to our observed effects of the inhibitory compounds on APD and ERP (Figure 2C, 3A, 3B, 3D), the spectral analysis of dominant frequencies revealed a reduction of the dominant frequencies for all conditions (Figure 6B). Although these effects were observed even for compounds with no effect on the number of spiral waves, changes were again most pronounced for compound #1 and #3.

### Effects of *I*_Kur_ inhibition on stability of rotors

**Figure 7:**
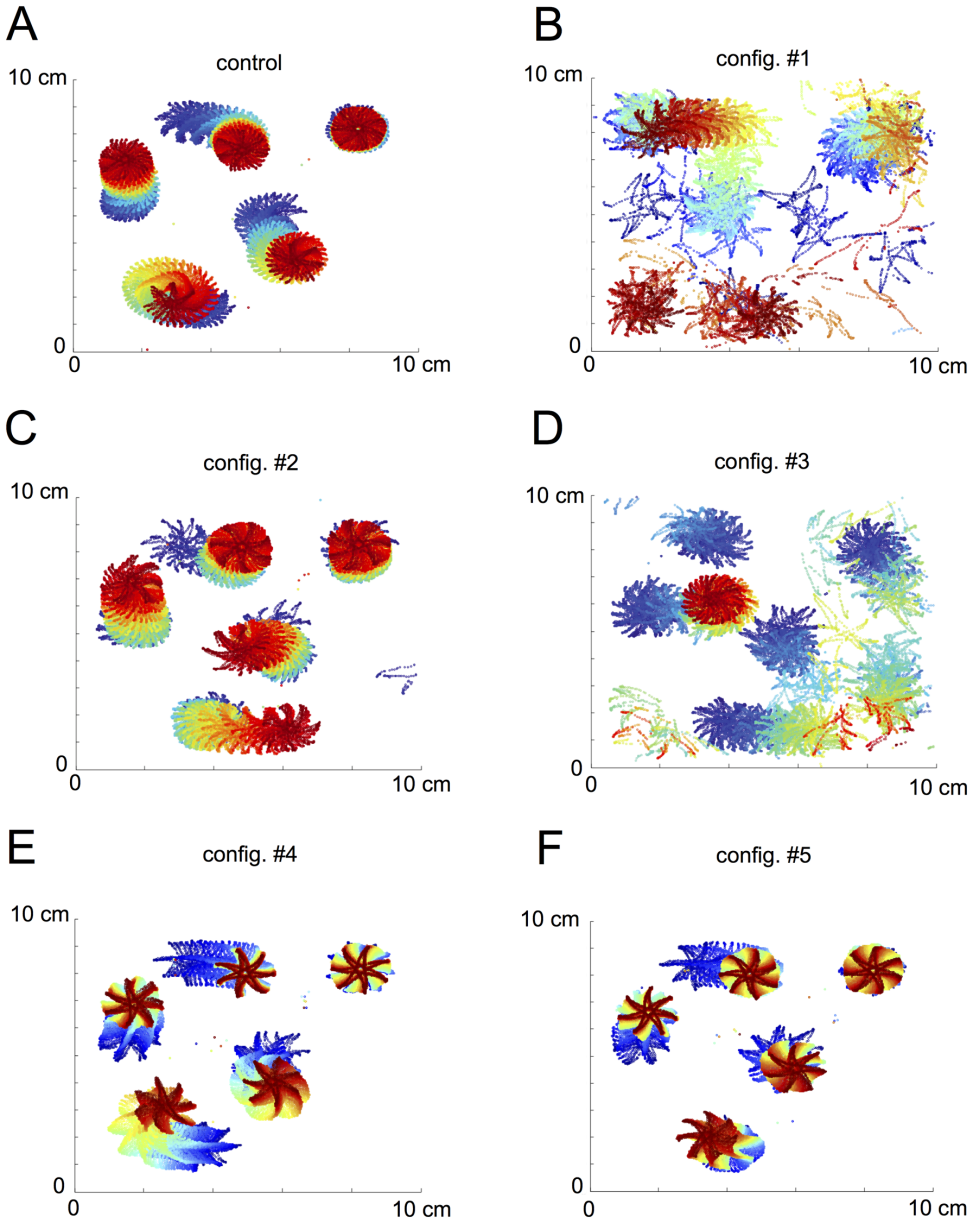
Effects of *I*_Kur_ inhibition on spiral tip meandering. Spatiotemporal arrangement of the spiral tips followed over a period of 30(color coded time scale: blue = early, red = late). Under control cAF conditions (A), five rotor centers were detected with high spatiotemporal stability. Similar results were obtained for the inhibitory compounds #2 (C), #4 (E), and #5 (F). However, when applying the compound #1 (B) or compound #3 (D), pronounced destabilization was observed paralleled by an extinction of rotors.

Besides being a powerful tool to easily assess the number of spiral waves, our automated detection algorithm also opens up the possibility to automatically track spiral-tip meandering. The spatial arrangement of the spiral waves under control conditions showed a rosette-like pattern with only minor movements of the rotor centers (Figure 7A; color coded time scale with blue representing early and red late time points). Similar results were obtained for compounds #2, #4, and #5 (Figure 7C, 7E, 7F). In these cases, no marked spatial instability of spiral tips could be observed. In contrast, compounds #1 and #3 induced a pronounced tip meandering resulting in the extinction of adjacent spiral waves. These results are in line with the pronounced effects of these two compounds on WL and might be best explained by a limitation of the availability of non-refractory space. Figure 7B and 7D depict spiral tip meandering under inhibition with compound #1 and #3.

## Discussion

Using a mathematical model of human atrial tissue, we analyzed the impact of time and voltage dependence of *I*_Kur_ inhibitors on basic electrophysiological properties as well as fibrillatory activity. Although inhibition of *I*_Kur_ failed to prolong the terminal phase of the AP in normal atrial cells, a marked prolongation could be observed in cAF cells. Similar results were obtained for the ERP in the one-dimensional simulations. As expected for pure potassium channel inhibitors, none of the model compounds resulted in a marked change of the CV. Using two-dimensional simulations of cAF tissue, the effects of the different kinetic properties on fibrillatory activity were analyzed. Under control conditions, a number of five stable spiral waves could be induced. Interestingly, a relevant reduction of the number of rotors could only be observed for two of the inhibitory compounds, both resulting in pronounced spiral-tip meandering. We further provide evidence that the observed anti-arrhythmic effect might be due to a limitation of the non-refractory space.

### Comparison with previous *in silico* models of AF

In our computational study, we used a modified Courtemanche-Ramirez-Nattel (CRN) model of atrial electrophysiology including electrophysiological remodeling. As reported previously by our group, electrical remodeling was introduced by reducing the maximum conductance of *I*_to_ and *I*_Ca,L_ to 65% and increasing the maximum conductance of *I*_K1_ by 110% [17]. These modifications result in an APD_90_ reduction from 300 ms to 129 which is concordant with experimental data derived from human atrial tissue [20]. Whereas current densities of *I*_to_, *I*_Ca,L_ and *I*_K1_ were altered similarly in the CAF2 case of Pandit's model, they further included a reduction of *I*_Kur_ by 50% [23]. For our experiments, we decided not to include a reduction of *I*_Kur_ in order to better visualize the differential impact of *I*_Kur_ blockade. However, in case of a 50% downregulation of *I*_Kur_, the control situation would be expected to be identical to compound #5 (tonic blockade) in terms of wavelength prolongation and wavelet count. Any further inhibition of the downregulated current by the test compounds would result in a further wavelength prolongation similar to our observed effects. However, considering the smaller impact of the downregulated *I*_Kur_ on repolarization, differences between the kinetic properties of the test compounds would be less pronounced. A significant difference between the models, however, relates to gap junction remodeling. In order to include AF induced alterations in connexin Cx40 expression, we decided to reduce the conductance of the monodomain equation by 30% [17], [21]. In our opinion, this reduction correlates well to the clinical data revealing a CV decrease of 20% in cAF atria [31]. Taken together, our changes resulted in a marked reduction of the cardiac WL defined as the distance traveled by the depolarizing wavefront during the functional refractory period [32]. As the number of reentrant circuits that fit into a certain area of atrial tissue depend on CV and hence WL, up to five stable spiral waves could be induced in our model. As discussed by Kneller et al. it is therefore tempting to speculate that in case of a larger two-dimensional sheet or reduced WL fibrillatory activity might more likely depend on multiple wavelet reentry than a primary rotor [26].

### Insights into antiarrhythmic effects of *I*_Kur_ inhibition

Data from computational studies provide interesting insights into antiarrhythmic mechanisms of different antiarrhythmic compounds. For example, Kneller and co-workers elegantly showed that pure sodium channel blockade results in AF termination by enlargement of the center of rotation, increased meandering of spiral tips and a reduction in the number of secondary wavelets [30]. By analyzing the effects of potassium current inhibition on wavelet stability in a computational model of AF, Pandit and colleagues found that inhibition of *I*_Kur_ and *I*_to_ but not *I*_Kr_ or *I*_Ks_ resulted in rotor termination [23]. However, the inhibition was simulated by a simple reduction of the maximum channel conductance without simulating any time or voltage dependence of block. Tsujimae et al. developed a mathematical model of voltage- and time-dependence of *I*_Kur_ inhibition [14]. Predicted from their model, an ideal anti-AF drug profile would exhibit fast onset (5 ms) and slow recovery (1000 ms) kinetics [14]. However, the validity of this prediction has not been tested yet. To test the hypothesis that antiarrhythmic properties of *I*_Kur_ inhibitors depend on kinetic properties of inhibition, we introduced the mathematical description of time- and voltage-dependence of block in our *in silico* model of cAF. Compound #1 represents the fictitious inhibitory profile recommended by Tsujimae et al. [14]. In contrast, compounds #2 (DPO-3) and #3 (DPO-1) relate to existing kinetic profiles of a recently discovered group of potent *I*_Kur_ inhibitors belonging to the group of diphenyl phosphine oxides (DPO) [11], [13], [22]. Compound #4 was selected arbitrarily in order to simulate the combination of slow onset and fast recovery kinetics, and compound #5 represents the heterozygous situation of a loss-of-function mutation without dominant negative effects. As expected from the work of Tsujimae, compound #1 and #3 resulted in the most pronounced prolongation of the wavelength (Figure 3D). It is well recognized, that spiral tip trajectories strongly depend on the wavelength and the excitability of the tissue (for review see [25]). Accordingly, the most pronounced spatial instability could be observed for compounds #1 and #3. From these results we conclude, that the prolongation of the wavelength results in a limitation of non-refractory space thereby resulting in spiral tip meandering and rotor extinction. The results from our computational studies are underpinned by experimental data obtained from the goat model of atrial fibrillation [33]. Analyzing antiarrhythmic effects of the class III antiarrhythmic compound AVE0118 on atrial fibrillation they show that the *I*_Kur_ and *I*_to_ blocker AVE0118 dose-dependently terminated persistent AF [33]. These results might come as expected, as AVE0118 has been shown to efficiently prolong the action potential in cardiomyocytes isolated from chronic AF patients [6]. Within this context compound #3, which is highly similar to the diphenyl phosphine oxide-1 (DPO-1), seems particularly promising and should be further evaluated. Taken together, drugs that efficiently prolong the wavelength in remodeled atrial cells seem to be best candidates for potent antiarrhythmics.

### Limitations

The computational model used in this study represents a simplification of the human situation in terms of 3D tissue geometry, anisotropic fiber arrangement, as well as structural and electrical heterogeneities: The analysis of spiral wave activity was carried out in a small two-dimensional tissue sheet. As a consequence, the influence of myocardial thickness as well as gradients in myocardial thickness could not be addressed. However, it is well recognized that the electrical dissociation between the epicardial layer and the endocardial bundle network increases the stability of AF by enlarging the area available for reentry [34]. This effect can be further aggravated by structural remodeling in the outer layer of the atria resulting in a reduction of electrical coupling thereby further stabilizing AF [35]. On the other hand, the existence of gradients in myocardial thickness seems relevant for the perpetuation of AF, as scroll waves have been shown to localize at the interface between thick and thin regions [36]. As a consequence, it is tempting to speculate that pharmacological effect of the test compounds might be less pronounced as in a realistic 3D model of atrial tissue, as well as in the human situation. Furthermore, it is well recognized that abrupt changes in fiber orientation, that can be found in the ostium of pulmonary veins, can result in activation delay and conduction block [37]. These regions might promote ectopic activity or microreentry thereby favoring the induction or perpetuation of atrial fibrillation. As a limitation of the model, the complexity of atrial fiber orientation was not included. Fibrillatory activity was followed over a time period of 30 seconds. Within this interval, two of the model compounds exerted relevant antiarrhythmic effects, whereas all others resulted in no marked reduction of spiral activity. However, none of the model compounds resulted in a complete termination of all rotors. This observation is somewhat contradictory to previously published effects of *I*_Kur_ blockade in *in silico* models of AF [23]. However, by reducing the CV, we presumably generated a substrate highly susceptible to reentrant activity thereby rendering the simple termination by ion channel inhibition more difficult.

### Conclusion

Taken together, we provide evidence that antiarrhythmic effects of *I*_Kur_ inhibitors are strongly dependent on kinetic properties of blockade. Especially compounds with slow recovery kinetics result in an effective prolongation of the wavelength thereby increasing the area of refractory space. In these cases chaotic meandering of spiral-tips leads to a reduction of reentrant circuits.
